# 

**Published:** 1891-10-17

**Authors:** 


### NOTICE TO CORRESPONDENTS.

All MS., Letters, Books for Review, and other matters intended for the Editor should be addressed The Editor, The Lodge, Porchester Square, London, W.

The Editor cannot undertake to return rejected M.S., even when accompanied by stamped directed envelope.

**Notice to Advertisers and Subscribers.**

All Advertisements, Orders for Copies of The Hospital, and Business Communications should be addressed to The Publisher, *not to the Editor*, The Hospital, 140, Strand, W.C.

The Cost of Subscription, post free, is as follows:—Per week, 1½d.; per quarter, 1s. 8d.; per half-year, 3s. 3d.; per annum, 6s. 6d. Foreign:—Per annum, 8s. 8d.; payable, in all cases, in advance.

**NOTICE.—The Index for Vol. IX.. (ending March, 1891) is on sale at the Office of this Journal, price 2½d., post free.**

### General Charities.

[This Column is devoted to an account of work of charitable institutions other than Medical Charities. The Editor will be glad to receive reports or news of work at 140, Strand. *Philanthropy* should be plainly written on the envelope.]

It is reported that there was a falling off amounting to £30,000 in the subscriptions received during the past year by **THE LIFEBOAT INSTITUTION**, a fact which is deeply to be regretted. That an Institution of such world-wide repute and of a national character, the management of which is acknowledged on all hands to be admirable, should have suffered so deeply is a matter for serious reflection. We fear that the public is too prone to be carried away by sensational appeals, and in the fervour of its emotion to forget old and tried institutions for the sake of untried schemes, the merits of which it does not stop to weigh. The emotional section of the public which is inclined to good deeds is thus too often guilty of withdrawing its support from meritorious societies in order to gratify a fleeting emotion rather than confer a benefit. This is not charity.

When so many able bodied men and women complain of their inability to get work to do it is not surprising that the blind find great difficulty in obtaining sufficient employment. Yet there are numerous trades suitable for the exercise of their somewhat narrowed powers, such as making mats, brooms, baskets, ropes, chair-caning, netting, and knitting. The Committee of the **SOUTH LONDON ASSOCIATION FOR ASSISTING THE BLIND** appeal to those who are interested in the welfare of the blind to remember this fact, and, if possible, to find employment for many who are willing to do their share of work. The Association confines its work principally to the improvement of the indigent blind living in the South of London, and we regret to notice that blind persons cannot obtain any benefit from the Association unless recommended by an annual subscriber. Those who are so recommended are instructed in trades, handicrafts, and such professions as are likely to be suitable.

The benefits of the pure oral system for the instruction of the deaf and dumb, though not yet, perhaps, fully acknowledged in this country, are such that the system is yearly gaining ground, and will ultimately prevail. The success attending the system of lip-reading in the Jews' Deaf and Dumb Home was such as to tempt the Baroness Meyer de Rothschild to extend its benefits to every race and creed by establishing a training college for teachers and a school in which deaf and dumb children of all classes and denominations should be taught to speak. The result of her efforts was the establishment of **THE ASSOCIATION FOR THE ORAL INSTRUCTION OF THE DEAF AND DUMB**, which has been doing good work for the past twenty years. To gain the end at which the Society aims, it is essential that good and well-trained teachers should be constantly employed, a point which the management keeps well in view. The Institution has for some few years been burdened with a debt, which was liquidated to a certain extent by a dinner held last year, and donations, which should be sent to the Secretary, Mr. Arthur G. Klugh, will undoubtedly be bestowed on a worthy object.

### Scraps and Gleanings.

A Hospital Sunday Collection is to be started in Londonderry.

No less than 145 different medical journals are published in Paris.

The Hospital Saturday and Sunday Collection at Poole has yielded £70.

The return of rabies is predicted in the metropolis. One or two cases have already occurred.

Ten thousand pounds is to be raised by loan for the erection of a fever hospital for Stockton.

The Leicester Infirmary has benefited to the extent of £1,678 through the Hospital Saturday collection.

The Bournemouth Hospital Saturday and Sunday Fund has been augmented by a collection of £592 this year.

The Hospital Saturday collection at Colchester has amounted to £169, which will be handed over to the Essex and Colchester Hospital.

The Abingdon Friendly Societies held their annual parade in aid of the Wallingford Cottage Hospital last week. The collection amounted to £18.

At the concerts held in aid of the General Hospital, at Birmingham, friends of the hospital subscribed to seat the Town Hall with arm-chairs.

The Nurses' Home, in connection with the Prince Alfred Hospital, will shortly be opened. The foundation-stone was laid by Lady Carrington.

St. Pancras Annual Ball, in aid of the Almshouses and the Female Orphanage, resulted in £90 being handed over to each of these meritorious institutions.

The annual report of the Lowestoft Hospital shows an increase in expenditure beyond its income of £105, owing to an increased number of in-patients.

The ceremony of opening the Printers' Almshouses at Wood Green by the Duchess of Albany, having been postponed from the 10th, will take place on October 17th.

It is hoped to establish a Cottage Hospital at Stourbridge shortly. The Governors of the Dispensary do not propose erecting new buildings, as only six beds are to be maintained.

Whilst we in England are viewing with alarm the increase of population, in Arkansas they are offering prizes for large families. A prize of 100 dollars was gained by a pair whose descendants numbered 93.

Sunday, September 27, was observed as the Harvest Thanksgiving at Southbourne-on-Sea, a small sea-side resort about four miles east of Bournemouth. The sum of £53 was handed to the hospital fund.

The Duke of Westminster has sent a cheque for £500 towards the funds of the Chester General Infirmary, being the total proceeds of the shilling entrance-fee charged to visitors for the privilege of seeing Eaton Hall.

Princess Louise visited Edinburgh last week, and presided at the meeting of the Council of the Scottish Branch Queen Victoria Jubilee Institute for Nurses, of which she is President. She presented the institute badge to 12 nurses.

M. Henri Monod, Director of the "Assistance" and of Public Hygiene, Paris, has made a very flattering report upon the beneficial result of the English sanitary laws in the prolongation of life, and the amelioration of the condition of the people.

At the last meeting of the Kingston Board of Guardians the statement relating to the condition of the old Infirmary was made. The report stated it to be in a dilapidated and insanitary condition. The erection of new buildings has been proposed.

The Northampton General Infirmary are about to carry out extensive repairs and improvements to the institution; £700 raised during the Jubilee year is available for the purpose. The annual report shows a favourable balance to the credit of the Infirmary.

A report on the dwellings of the poor states that numerous insanitary houses have been found in Finsbury, Holborn, Hammersmith, Poplar, Mile End, Lewisham, Westminster, St. Pancras, and elsewhere. Several houses in Southwark are ordered to be demolished.

Mr. M. Carteighe distributed the prizes to the successful students of the school of the Pharmaceutical Society of Great Britain last week at Bloomsbury Square. The school will celebrate its jubilee next year. The first prizeman was H. Garnett, who received three silver medals.

At the last monthly meeting of the Grimsby and District Hospital it was mentioned that a little girl, whose mother had been a patient in the hospital, had collected £1 9s. on the seashore in aid of the hospital. It is pleasant to hear of such a substantial return for benefits received.

Nearly £29,000 was received as the result of the chairmanship of the Earl of Lathom at the last festival of the Freemasons' Boys' School, and in consequence of that great success the quarterly court of subscribers yesterday admitted five additional boys to the benefits of the institution.

The *Globe* states that for some time past the handkerchief trade has been languishing. Since the advent of autumn [illegible] this is no longer the case. Sufferers from catarrhal affections are invited to take consolation from the thought that they are giving employment to a deserving class of the community.

Twenty-eight skeletons were uncovered during some excavations near the depot of the Artillery Volunteers at Southover, Lewes, within an area of about 130 ft. by 50 ft. As the skeletons comprise those of women as well as of men, it is concluded that the site was not that of a battle-field, but of a place of burial.

A French chemist, in search of an antiseptic, turned various microbes into the densest Dead Sea water that had ever been fetched to his laboratory. The diphtheria, measles, scarlatina, small-pox, and others, all died but two, with which in 48 hours the fluid was alive. They were the microbes of tetanus and gangrene.

Last week the Archbishop of Armagh, performed the ceremony of blessing the foundation stone of the Shiel Hospital, Ballyshannon, an hospital which is to perpetuate the name of the late Dr. Shiel, of Ballyshannon, who made a bequest of about £6,000 for that purpose. Mr. W. H. Byrne, architect of Suffolk Street, has designed a building suitable in every way.

### The Student of Medicine.

[All communications for this Supplement should be addressed to The Editor of The Hospital, at 140, Strand, with the words, "Students' Column," written in the left hand top corner of the envelope.]

### APPOINTMENTS.

At the Royal Infirmary, Edinburgh, K. M. Douglas, M.D., F.R.C.P. Edin., has been appointed surgical registrar; and A. L. Gillespie, M.B., C.M. Edin., medical registrar. At St. Mary's Hospital, Manchester, W. J. Kerr, M.B., B.Ch.Vict., M.R.C.S., L.R.C.P., has been appointed resident medical officer. At the Great Northern Central Hospital, W. A. Malcolm, M.B., C.M. Edin., has been appo[illegible]ted anæsthetist. At the London Temperance Hospital, Gerald Mansfield, M.B., C.M. Edin., has been appointed senior resident med[illegible] officer. At the City Fever Hospital, Edinburgh, Claud Muirhead, M.D., F.R.C.P.Edin., has been appointed consulting surgeon. At the Ancoats Hospital, Manchester, Thomas Porter, M.B., B.Ch. Vict., has been appointed house surgeon. At the Royal Hospital for Children and Women, A. Marmaduke Shield, M.B., B.C., F.R.C.S., has been appointed assistant surgeon.

### VACANCIES.

The following vacancies are announced this week, the amount of salary in each case being given after the name of the institution:

**Resident Medical Officers.**

Brompton and Knightsbridge Dispensary, Medical Officer.

Cheltenham General Hospital, House Surgeon—Salary £80, and board, &c.

Eastern Counties Asylum for Idiots, Colchester, Resident Medical Attendant—Salary £100, and board, &c.

North-West London Hospital, Resident Medical Officer—Salary £50, and board, &c.

Paddington Green Children's Hospital, House Surgeon—Salary £50, and board, &c.

Royal Isle of Wight Infirmary and County Hospital, Ryde, House Surgeon and Secretary—Salary £60, and board, &c.

Salford Union, Assistant Medical Officer—Salary £130.

Warrington Infirmary and Dispensary, Junior Resident Medical Officer—Salary £100, and board, &c.

**Non-Resident Medical Officers.**

City of London Hospital for Diseases of the Chest, Assistant Physician.
Hastings, St. Leonards, and East Sussex Hospital, Honorary Assistant Physician.
Salford Royal Hospital, Honorary Surgeon.

### SCHOLARSHIPS AND PRIZES.

**University of Aberdeen, Summer Session, 1891.**

OPERATIVE SURGERY.

Honours List Second Attendance.

Bronze Medal and Highest Honours Certificate.—A. Stables, 90·1.
Highest Honours Certificate.—M. W. Renton, 75·1.
Honours Certificates.—H. M. Mitchell, 71·4; W. H. de Silva, 66·9; C. G. M'Gregor, 63·1; A. W. Thomson, 62·1.

First Attendance.

Bronze Medal and Highest Honours Certificate.—J. Matheson, M.A., 77·8.
Honours Certificates.—A. W. Mackintosh, M.A., 74·5; A. A. Moore, 74·4; W. Hector, 73·1; F. A. Gill, 70·3; G. J. A. Watson, M.A., 68·9; A. Duncan, 67·6; G. Sturrock, 67·1; A. Brown, M.A., 66·8; J. Ingram, 66·6; P. M. Muttukumaru, 66·3; H. Fraser, 65·5; A. Ogston, M.A., 63·4; P. Howie, 63·3; G. Marr, 60·5; W. Thomson, 60·3; C. K. Morgan, 60.

PRACTICAL PATHOLOGY.

First Class.—J. T. West (medal), 93 per cent.; A. Grant (medal), 83; A. Fraser, 82; G. Savege, 76; A. D. Milne and J. Rust, M.A., 75.

Second Class.—W. Dunn, W. R. Pirie, M.A., and W. J. S. Ewan—equal, 72; R. Ferguson, 69; J. W. Grant, 68; R. Kerr, 62; W. Ross 57; G. Geddes, 55, J. Wallace, 52; C. Grant, 51.

### ATHLETICS.

**Football.**—Association (October 9).

*London Hospital* were beaten by *Foxes* at Edmonton by five goals to two:

Rugby (October 9).

*Bartholomew Hospital* beat *Civil Service* at Balham by a goal and three tries (eleven points) to nil.
*Middlesex Hospital* beat *Ealing* at Ealing by one goal to nil. Try by Marshall.
*St. Mary's Hospital* were beaten by *Old Paulines* at Chiswick by a goal to nil.
*St. Thomas's Hospital* drew with *Old Leysians* at Stamford Bridge. Score at half-time, nil to nil. Result, one goal each. (Five points.)
*Westminster Hospital* beat the *Royal Naval School* at Eltham by one goal and three tries to one try.

